# Stress Analysis of a Class II MO-Restored Tooth Using a 3D CT-Based Finite Element Model

**DOI:** 10.1155/2012/657519

**Published:** 2012-07-14

**Authors:** Yiu Pong Chan, Chak Yin Tang, Bo Gao

**Affiliations:** ^1^Department of Industrial and Systems Engineering, The Hong Kong Polytechnic University, Hung Hom, Kowloon, Hong Kong; ^2^Department of Prosthodontics, College of Stomatology, The Fourth Military Medical University, Xi'an 710032, China

## Abstract

A computational method has been developed for stress analysis of a restored tooth so that experimental effort can be minimized. The objectives of this study include (i) developing a method to create a 3D FE assembly model for a restored tooth based on CT images and (ii) conducting stress analysis of the restored tooth using the 3D FE model established. To build up a solid computational model of a tooth, a method has been proposed to construct a 3D model from 2D CT-scanned images. Facilitated with CAD tools, the 3D tooth model has been virtually incorporated with a Class II MO restoration. The tooth model is triphasic, including the enamel, dentin, and pulp phases. To mimic the natural constraint on the movement of the tooth model, its corresponding mandible model has also been generated. The relative high maximum principal stress values were computed at the surface under loading and in the marginal region of the interface between the restoration and the tooth phases.

## 1. Introduction

Ethical concerns limit laboratory studies on living subjects, and advanced digital imaging technology has opened up new alternative possibilities in dentistry. The computational simulation method takes a more important position in both clinical and therapeutic applications in the dental industry [1]. Usage of virtual models offers an alternative method of investigation, and costs can also be reduced for in vivo and in vitro experiments.

In dental research, CT scanning is the most frequently used high resolution imaging technology. To conduct CT scans, the targeted object needs to be exposed to a certain amount of ionizing radiation in which the absorbed radiation is detected and imaged later on. A series of sliced 2D images, depicting a density map of the scanned object, can be obtained. Piling these images creates a 3D description of the scanned area. The development of CT technology has been used to study and quantify the morphology of bone [2, 3], to nondestructively evaluate porous biomaterials [4], and to investigate the architecture of scaffolds [5, 6]. The offering of reasonably high resolution is the main reason for using CT as imaging technology in the field of hard tissue engineering. Contrast segmentation is a technique used for tissue differentiation, in which the grayscale of the layered contour images is determined by the tissue density. Living tissues involve complex shapes, for example, teeth and mandibles that are possible to be digitized using CT scanned images to allow accurate measurement of small changes of irregularities [7]. Ulusoy and Darendeliler [8] have evaluated the effects on the stress distribution with different types of Class II activators using a mandible FE model constructed using CT-scanned images. Bujtár et al. [9] have digitized the mandibles of patients of different ages using CT-scanned images, and performed analyses with the simulation of supra normal chewing forces. Gao and Chae [10] have proposed a tooth segmentation method to visualize a tooth from CT images so as to provide assistance for dentists performing orthodontic surgery and treatment.

With the availability of the advanced imaging technology, significant progress has been made in the past decade. Ausiello et al. [1] have constructed a 3D premolar model through digitizing a plaster tooth model with manual operations of the literature data related to the volume of dentin and enamel. Cattaneo et al. [11] have attempted to build up a model of maxilla with two molars from a living object and to investigate the distribution of stress under occlusal forces, in which the molar parts are assigned with uniform mechanical properties. Ichim et al. [12] have modeled the fracture behavior of a restoration using a 2D FE model constructed using CT-scanned images with the consideration of internal tooth structure. Magne [13] has demonstrated the usage of Boolean operations to build up a 3D tooth model with desired restoration morphologies using CT images. In contrast, Lin et al. [14, 15] have used a 3D tooth model constructed manually, according to the literature data describing the morphology of teeth, to determine the relative contribution of changes in cavity dimensions and various factors on the biomechanical response of a restoration during oral temperature changes. In 2010, Li et al. [16] constructed a 2D FE model from scanned images for optimizing the shape of the restoration cavity. Li et al. [17] have used scanned images to construct a 2D FE model for the simulation of debonding and fracture of a restored tooth.

Understanding how mastication loading is spread over a restored tooth is crucial to both clinical practitioners and material scientists [18]. Miura et al. [19] have stated that periodontal tissue, such as in the mandible, which constrains the location of the tooth, plays an important role for failure prediction in dental research. The aim of the present study is to develop a computational method for the stress analysis of a restored tooth so that experimental effort can be minimized. The objectives of this study therefore are to develop a method to create a 3D FE assembly model for a restored tooth and its corresponding mandible based on CT images; and to conduct stress analysis of the restored tooth using the 3D FE model established. In this study, the tooth model is a triphasic one, consisting of enamel, dentin, and pulp phases, which gives rise to a more realistic simulation to estimate the stress distribution of the tooth and the restoration. Moreover, the proposed method offers a computational scheme assisting dentists to design a more effective tooth repair strategy.

## 2. Methodology

**Figure 1:**
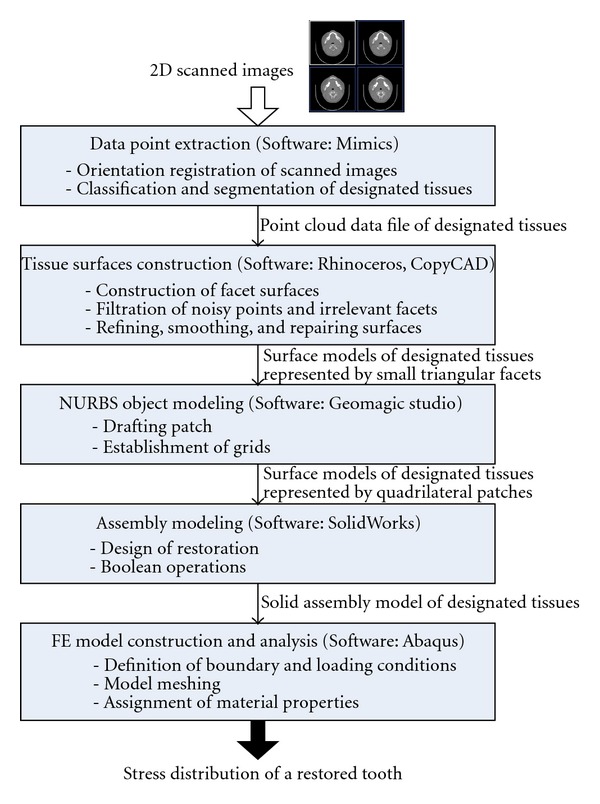
The five-procedure method in generating a 3D CT-based FE model from 2D scanned images.

In this study, a 3D FE model of a restored tooth with a mandible is created from CT-scanned images following the five stages shown in Figure 1. The first stage is to extract the 3D raw data representing the designated tissues in the format of a point cloud from 2D scanned images. The second stage aims to create surface models of the enamel, dentin, pulp, and mandible using tiny triangular facets based on the extracted point cloud data. The purpose of the third stage is to represent the surface models using tiny facets to bigger quadrilateral patches, which facilitates performing modifications on the generated model in the next stage. The fourth stage aims to transform the surface models into a solid assembly model comprising a tooth and a mandible submodel. In addition, a Class II mesioocclusal (MO) restoration structure has been incorporated into the tooth sub-model using the CAD tools. The last stage aims to build up the FE model based on the solid assembly model and undertake computational analysis of the stress distribution on a restored tooth under mastication loading.

### 2.1. Data Point Extraction

This procedure is to align the discrete 2D scanned images into one 3D coordinate system. In addition, it is aimed at extracting the geometric data of the designated tissues from the processed images and storing them in a point cloud format.

**Figure 2:**
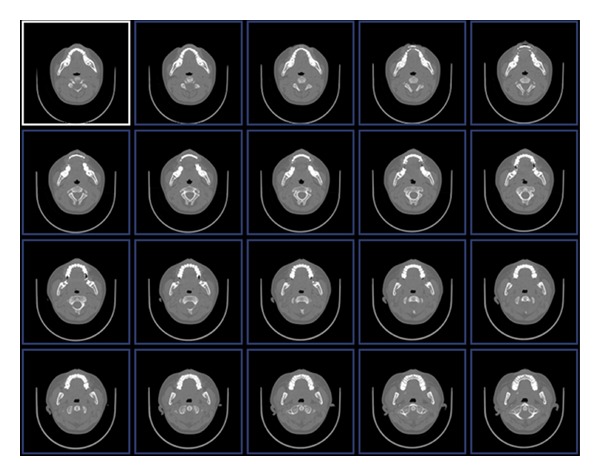
Part of the organized CT images in the top view.

In this study, the primary input is the CT scan images. A CT scanner, Bright Speed ct 99 (GE Medical System), was used to obtain the skull images of a 20 years old male. The voltage used was 120 kV, and the current was 300 mA. The slice thickness was 1.25 mm and the pitch ratio was 0.875 : 1. The scan option was 0.8 s, and a total of 180 images were obtained in 60 s. The sets of anatomical images acquired from different 2D perspectives (i.e., top, front, and side) were stored in the DICOM format. The sliced images in the top view were organized in sequence and some are shown in Figure 2.

**Figure 3:**
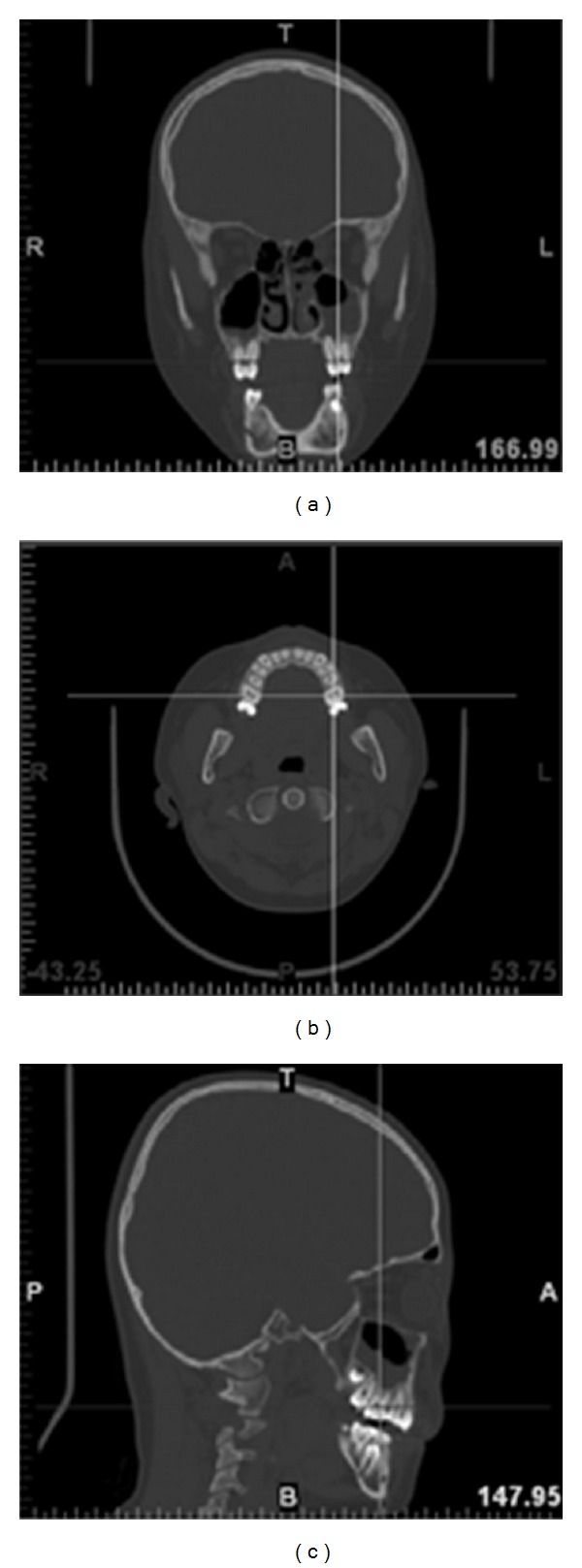
Orientation parameters registration, top T, bottom B, left L, right R, anterior A, and posterior P, for (a) front view images; (b) top view images, and (c) side view images.

The organized images are then imported into the software package, Mimics, Version 10.01, Materialise, Leuven, Belgium, to perform the orientation registration process. The process involves the assignment of orientation parameters, top (T), bottom (B), left (L), right (R), anterior (A), and posterior (P), to each anatomical image (Figure 3). In addition, the separation distance for each consecutive scanned image was also specified. In this study, the distance was set as 1.25 mm.

**Figure 4:**
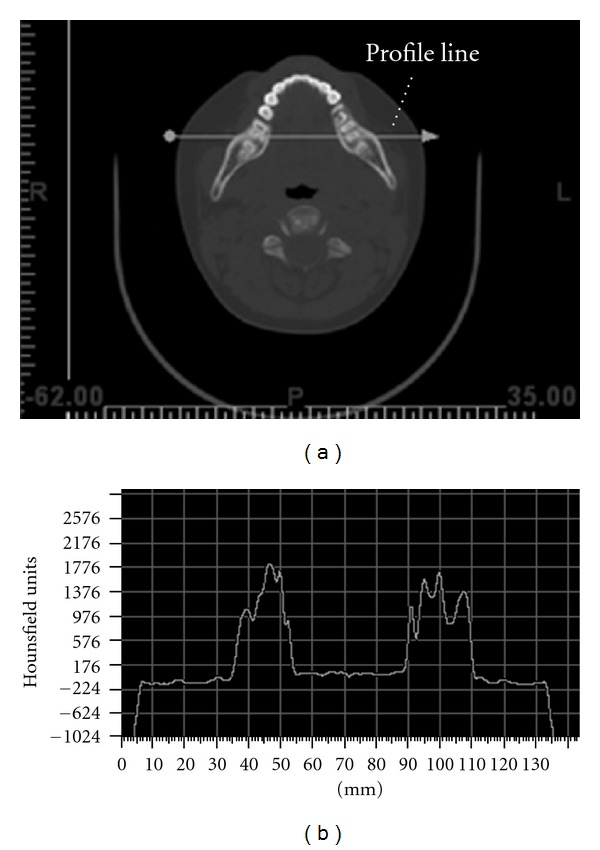
(a) A sliced image with a profile line (about the region with teeth and mandible); (b) the corresponding variations of HU value along the profile line.

**Figure 5:**
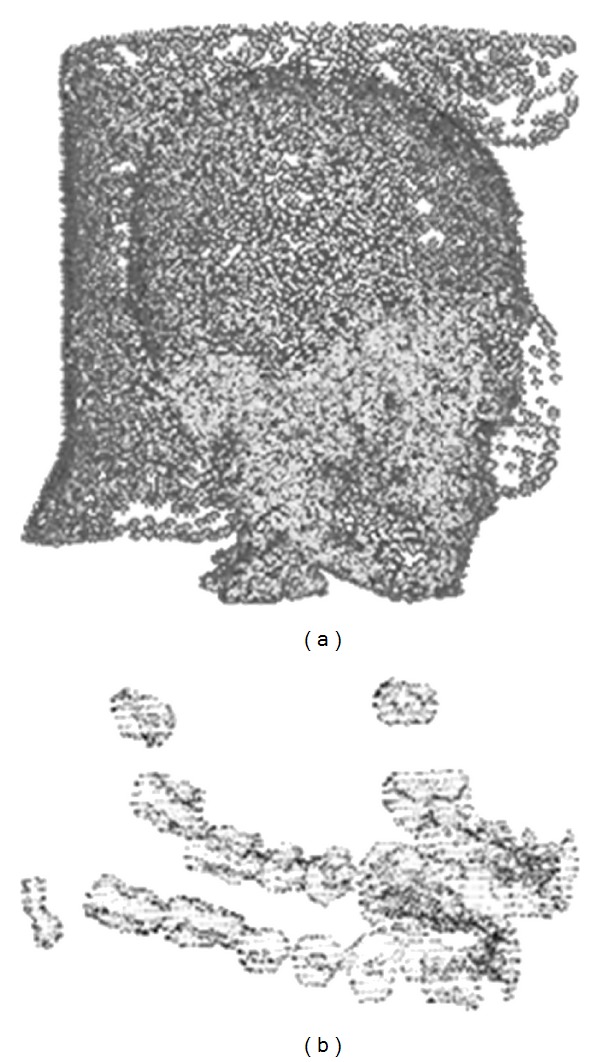
The preliminary obtained geometric point cloud data for (a) mandible, dentin, and pulp descriptions; (b) enamel description.

Data on the designated tissues has been extracted through classification and segmentation processes. Each 2D image slice can be regarded as a pixel map of the radiation attenuation tissue coefficient. The level of attenuation can be measured in Hounsfield units (HUs). HU is a normalized unit whereby water has a value of 0 while air has a value of −1024. The classification process aims at identifying the HU value describing the targeted tissue in each scanned image, while segmentation is a process of identifying the HU value representing the inner and outer surface boundaries of the tissue. To do so, a profile line was firstly constructed on a scanned image. The upper and lower limits in terms of HU values describing the designated tissues were determined from the profile map.  Figure 4 shows a slice of a scanned image and the corresponding variations of HU along the profile line. Through the classification and segmentation functions of mimics, the geometric data of the desired anatomical tissue can be preliminary extracted. To extract the geometric data for the mandible, dentin, and pulp, an HU value within the range of 176–2176 has been used while 2042–3071 has been defined for enamel.  Figure 5 presents the preliminary geometric descriptions, in which the descriptions are transformed and presented in point cloud format.

### 2.2. Tissue Surfaces Construction

The purpose of this procedure is to generate surface models of the designated tissues, which are free from imperfections, from the point cloud data through filtration, smoothing, refining, and repairing processes.

**Figure 6:**
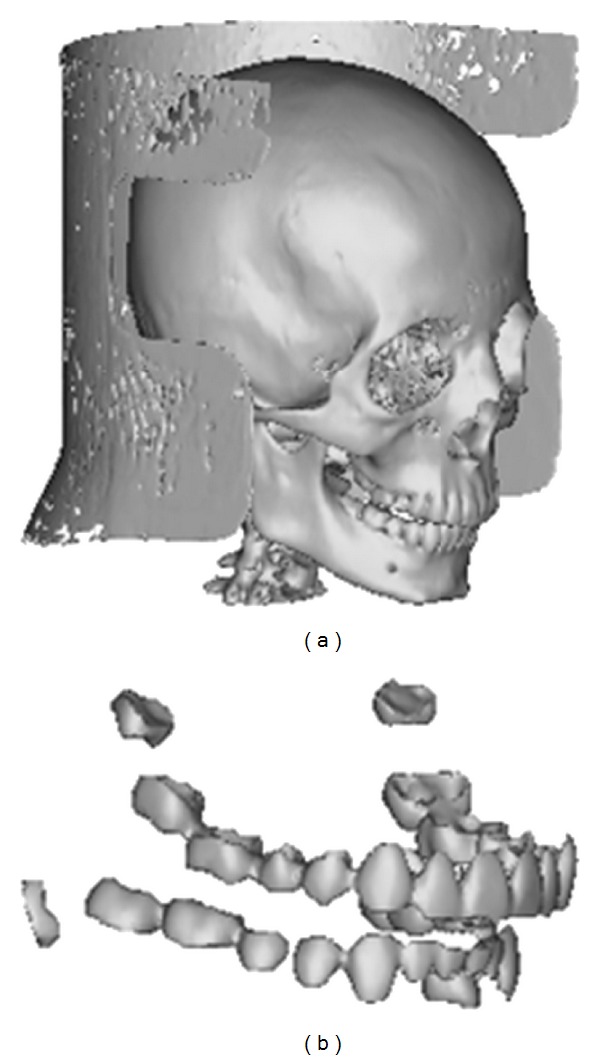
The surface models created through the polygon/facet creation process.

At first, the point cloud data acquired from the previous procedure underwent a polygon/facet creation process, and the software package, Rhino, Version 3.0, Rhinoceros, Seattle, USA, was employed. Facilitated by the software, facets were formed and joined. Surface models were then created as shown in Figure 6. Since the classification and segmentation processes (conducted in the previous procedure) are performed throughout the whole set of anatomic images, surfaces of nondesignated parts having similar HU description values were also generated. In addition, polygon/facet creation was a blind process. Irrelevant facets and noisy points are inevitably formed which could hinder the conversion to the solid model later on. Therefore, a filtering process was undertaken to erase the non-designated/unwanted parts as well as the noisy points and irrelevant facets.

**Figure 7:**
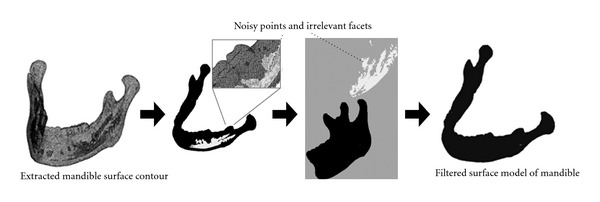
An illustrative example of separating noisy point data and irrelevant facets from the designated mandible tissue surface.

The filtration process was performed with the aid of the embedded clustering function from Rhinoceros. As demonstrated in Figure 7, the surface contour of the mandible was extracted and separated from the noisy points and irrelevant facets. Similarly, the surface contours of enamel, dentin, and pulp were also extracted and filtered.

Surface refinement and smoothing processes were then performed using the software package, CopyCAD, Delcam, Taipei City, Taiwan. Surface refinement involves the detection and deduction of sharp facet triangles and self-intersecting facet triangles. Such action can minimize the chance of error in creating the mesh model in the later procedure. A surface smoothing process was then carried out, which performs a similar operation through the action of modifications rather than deductions. This process involves the definition of a smooth factor (SF) value (a real number ranging from 0 to 1) and an iterative parameter (IP). In this study, the SF and IP parameters were given values of 0.1 and 3, respectively.

**Figure 8:**
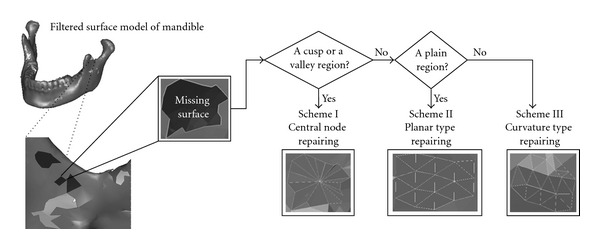
Surface repairing process involved in the tissue surface construction and processing step.

Surface repairing was also performed in this procedure, dealing with repairing imperfections, such as small holes and big cavities, in the designated surfaces. Such process was carried out through one of the three schemes, shown in Figure 8, based on the selection criteria. This procedure is important in constructing a closed-surface model which is a must condition for the solid model construction process in the later procedure.

### 2.3. NURBS Object Modeling

**Figure 9:**
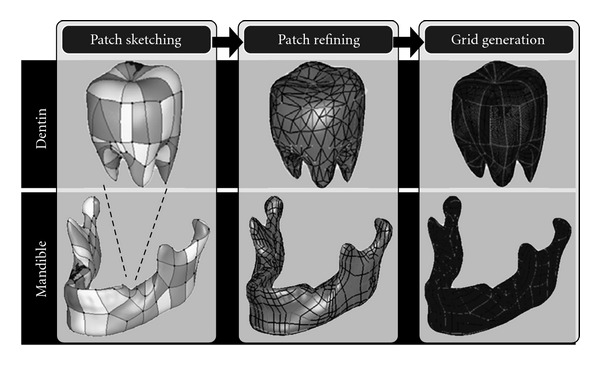
Processes involved in the NURBS object modeling procedure.

This procedure aims to represent the facet surfaces of the designated tissues using the non-uniform rational basis spline (NURBS) mathematical model. NURBS representing surfaces allows better performance in designing and creating restorations using CAD tools in the next stage. Patch sketching, patch refining, and grid generation are the three processes involved in converting the facet surfaces into NURBS representing surfaces. After the three processes, the morphology of the imported surface models can be kept but is represented using quadrilateral patches.  Figure 9 illustrates the processes involved in creating the NURBS representing surfaces of dentin and mandible. This was done using the software package, Geomagic Studio, Version 10, Geomagic, Morrisville, USA.

### 2.4. Assembly Modeling

This procedure is aimed at transforming the NURBS surface model into a solid model. Using the CAD tools, a conceptual research idea can be substantiated virtually and established interactively according to the freeform geometry of living tissues. In this study, a Class II MO restoration structure has been incorporated virtually into a tooth model. 

Firstly, the imported NURBS surface models of the mandible and tooth underwent a geometric diagnosis process using the CAD software package, SolidWorks Office Premium, Version 2007, SolidWorks, MA, USA. If a model is detected in an opened state, it needs to be reworked from the “Tissue Surfaces Construction” procedure, where the surface repairing process can be performed. If a model is identified to be in a closed state, a solid model can be generated directly.

**Figure 10:**
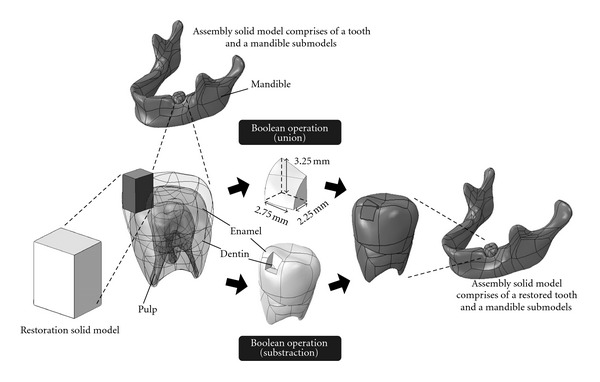
Processes involved in the assembly modeling procedure.

Facilitated with the CAD tools, Boolean operations were performed for the construction of a restored tooth.  Figure 10 demonstrates the Boolean subtraction process for creating a restoration cavity on the tooth model. Additionally, the Boolean union process was executed to trim off and customize the Class II MO restoration model according to the geometry of a tooth surface with dimensions given by Yaman et al. [20]. In addition, to simulate the mastication process, a ball with a diameter of 6 mm was constructed to apply occlusal loading to the tooth model [14]. The desired assembly model with a restored tooth and a mandible submodels was then prepared.

### 2.5. FE Model Construction and Analysis

This procedure aims to further develop the assembly solid model from the previous procedure to a FE model and involves the definition of the loading and boundary conditions, as well as meshing the model using finite elements. After assigning material properties to different phases of the model, analysis is undertaken to predict the stress distribution of a restored tooth under mastication loading.

**Figure 11:**
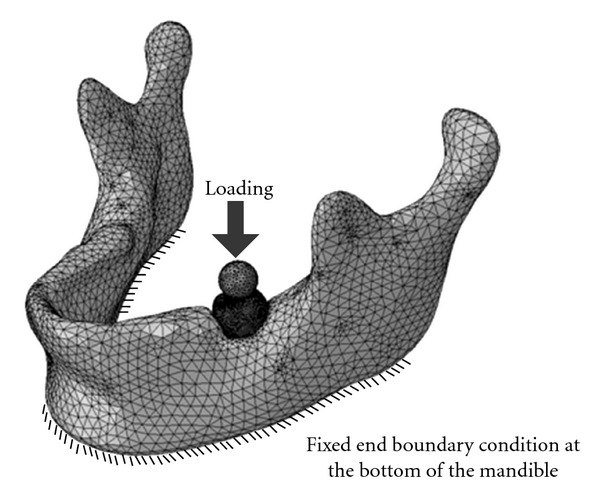
The meshed model (comprises of a restored tooth and a mandible) with description of loading and boundary conditions.

As shown in Figure 11, the assembly model of a mandible with a restored tooth was meshed using 3D tetrahedral solid elements (C3D4). This was done using the CAE software package, ABAQUS, Version 6.8, Dassault Systèmes, Simulia, China. To simulate the mastication loading, a spherical morsel with diameter of 6 mm was used having impact velocity of 0.03 mm s^−1^ exerted on the restoration [15, 20]. Simplified boundary conditions were applied, and the base of the mandible model was fixed. The material properties for the morsel, restoration, enamel, dentin, pulp, and mandible phases of the model were assigned as stated in Table 1.

## 3. Results and Discussions

To construct the tissue surfaces, there involves the definition of SF and IP values. The SF value indicates the importance of the local geometry. If the local geometry is important, the value of the SF will be set to a small value, which indicates that the corresponding smoothing action is limited. If a large value of SF (close to 1) is set, the position of the nodes of the facet triangle sets would be rearranged and the newly assigned position is mainly determined by the position of the other points of the facet triangles in the neighborhood. The number of cycles for smoothing depends on the defined IP. The definition of IP needs to be exercised with caution and to avoid exaggeration. If too many cycles of smoothing are performed, the imported 3D object would be turned into a sphere-like object. Suitable iteration cycles can maintain the morphology of the part of interest while reducing computational loading when carrying out simulation analysis.

**Figure 12:**
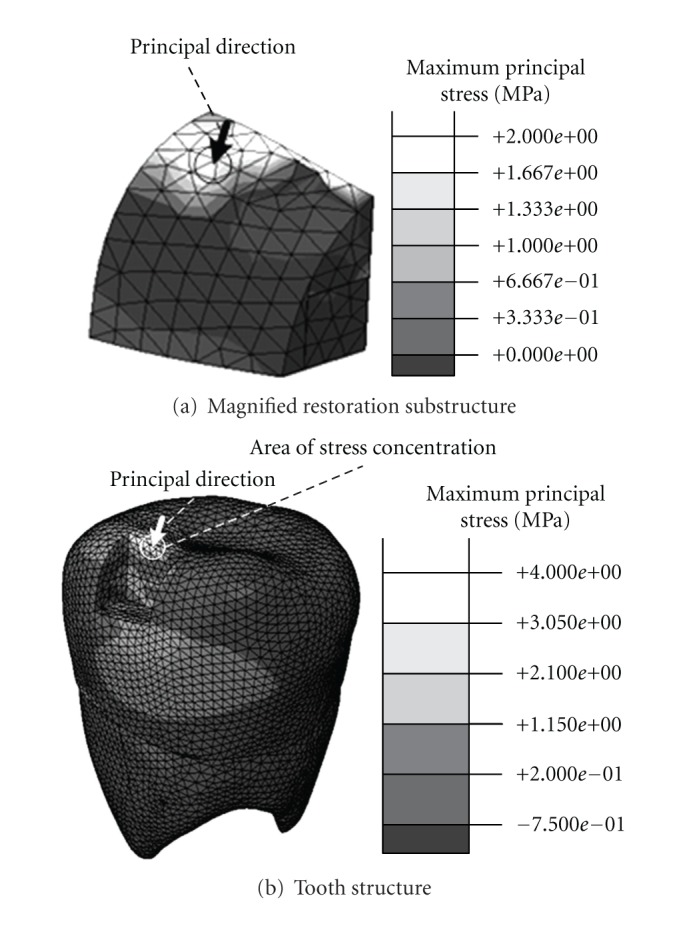
The predicted maximum principal stress of the restoration and tooth phases under the simulated mastication loading.

Budyn and Hoc (2007) stated that damage cracks can be initiated perpendicularly to the maximum principal stress direction, so the maximum principal stress of the model has been computed.  Figure 12 presents the stress contours in the restoration and tooth phases under the simulated mastication loading. Concerning the restoration phase, most of the loading was sustained at the surface having direct contact with the morsel and in the marginal region of the interface between the restoration and the tooth phases. Regarding the tooth phase, concentrations of stress were observed in the marginal region near to the loading. The principal direction of the element having the highest maximum principal stress value is also shown in the figure, which highlights the possible direction for the onset of damage. The predicted stress distribution can provide the important information to formulate a tooth repair strategy avoiding the failure of dental restoration before conducting actual repairs.

## 4. Conclusions

In this study, a viable alternative method involving the application of commercially available software packages has been used to create a 3D FE solid assembly model based on 2D CT images. A Class II MO restoration has been incorporated virtually into the tooth model. Computational analysis of the stress distribution on a restored tooth under mastication loading has also been conducted. With appropriate modifications, the generated assembly model can be extended for further computational investigations of various classes of restorations and cavity designs.

## Figures and Tables

**Table 1:** Mechanical properties of the morsel, restoration, enamel, dentin, pulp, and mandible phases.

	Elastic modulus (GPa)	Poisson's ratio
Morsel [17]	210	0.30
Restoration [21]	10.7	0.28
Enamel [21]	80.4	0.33
Dentin [21]	19.9	0.31
Pulp [21]	2	0.45
Mandible [21]	14.7	0.30
